# A general method for selection of riboflavin-overproducing food grade micro-organisms

**DOI:** 10.1186/1475-2859-5-24

**Published:** 2006-07-18

**Authors:** Catherine M Burgess, Eddy J Smid, Ger Rutten, Douwe van Sinderen

**Affiliations:** 1 Department of Microbiology and Biosciences Institute, National University of Ireland Cork, Western Road, Cork, Ireland; 2 NIZO Food Research, PO Box 20, 6710 BA Ede, The Netherlands; 3 Alimentary Pharmabiotic Centre, Biosciences Institute, National University of Ireland Cork, Western Road, Cork, Ireland; 4 current address: Dept of Food Safety, Teagasc-Ashtown Food Research Centre, Ashtown, Dublin 15, Ireland

## Abstract

**Background:**

This study describes a strategy to select and isolate spontaneous riboflavin-overproducing strains of *Lactobacillus *(*Lb*.) *plantarum*, *Leuconostoc *(*Lc*.) *mesenteroides *and *Propionibacterium *(*P*.) *freudenreichii*.

**Results:**

The toxic riboflavin analogue roseoflavin was used to isolate natural riboflavin-overproducing variants of the food grade micro-organisms *Lb. plantarum*, *Lc. mesenteroides *and *P. freudenreichii *strains. The method was successfully employed for strains of all three species. The mutation(s) responsible for the observed overproduction of riboflavin were identified for isolates of two species.

**Conclusion:**

Selection for spontaneous roseoflavin-resistant mutants was found to be a reliable method to obtain natural riboflavin-overproducing strains of a number of species commonly used in the food industry. This study presents a convenient method for deriving riboflavin-overproducing strains of bacterial starter cultures, which are currently used in the food industry, by a non-recombinant methodology. Use of such starter strains can be exploited to increase the vitamin content in certain food products.

## Background

The term "nutraceutical" was first coined in 1989 as a contraction of the words "nutrition" and "pharmaceutical", and refers to a food compound that not only supplements the diet but also aids in the prevention and/or treatment of disease and/or disorder [1]. Similarly, functional foods contain at least one component, whether it be a nutrient or not, that affects a target function of the organism in a specific, positive way, thereby generating a physiological effect beyond its nutritional value [2]. Functional food ingredients include probiotics, prebiotics, vitamins and minerals, and can be found in such diverse products as fermented dairy products, sports drinks and chewing gum [3,4]. Interest in and acceptance of functional foods is gaining impetus, for a number of reasons that include changing consumer demands and social attitudes, scientific evidence of the health benefits of particular ingredients and commercially driven interests to add value to existing products. Consumer awareness on nutrition raises the demand for healthy food options, ideally delivered in a convenient form [5]. Functional foods have a significant and growing global market with recent estimates indicating up to a $50 billion annual share [4].

Since Metchnikoff first theorised that fermented milk products provided health benefits, including longer life expectancy, these products have been viewed as "healthy" by consumers [6]. In many modern societies fermented dairy products make up a substantial proportion of the total daily food consumption. Lactic acid bacteria (LAB) are an industrially important group of micro-organisms used all over the world for centuries in a large variety of food fermentations, such as those of meat and vegetables, but undoubtedly with the major application in the dairy industry. LAB have been shown to be ideal cell factories because the biosynthetic capacity and metabolic versatility of these bacteria is generally quite limited, their physiology is relatively simple, while their energy metabolism and biosynthesis processes are almost completely separated [7]. It has therefore been possible to exploit LAB for the production of many nutraceuticals [8]. The dairy propionibacteria, especially *P. freudenreichii *ssp. *shermanii*, are the main ripening flora in Swiss-type cheeses, where they play a critical role in the development of the characteristic flavour and "eye" production [9]. Propionibacteria are also used for the production of vitamin B12 [10] and there is increasing interest in their potential use as probiotics [11]. Considering the current widespread use of LAB and propionibacteria in the food industry, coupled with consumer demand for healthier foods, the potential to use these food grade microorganisms as "vitamin factories" was investigated.

Riboflavin is a water-soluble vitamin produced by plants and many micro-organisms. However, this biosynthetic capability is lacking in higher animals and they must therefore obtain this essential nutrient from their diet. Riboflavin is the precursor of the enzyme cofactors FMN and FAD, which are vital in many of the body's enzymatic functions for the transfer of electrons in oxidation-reduction reactions. Riboflavin deficiency is most commonly seen in developing countries [12], among the elderly [13], and in chronic alcoholics [14]. Clinical symptoms of riboflavin deficiency are rarely seen in developed countries but the subclinical stage of deficiency, characterised by a change in biochemical indices, is seen in a significant portion of the population of these nations as exemplified by Ireland [15]. Riboflavin deficiency mainly manifests itself clinically in the mucocutaneous surfaces of the mouth, through the occurrence of cracks at the corners, and inflammation of the lips and tongue [16], but deficiency is also associated with vision deterioration and growth failure. In recent years the vitamin has been found to be effective in the treatment of migraine [17], malaria [18] and Parkinson's disease [19]. Riboflavin is commonly obtained in the diet from meat, eggs, fortified cereals and green leafy vegetables, in addition to dairy products, which contribute most significantly to riboflavin intake [20].

Riboflavin has been traditionally synthesised for food and feed fortification by chemical means but in more recent years biotechnological processes employing various bacteria, yeast and fungi have been found to be commercially competitive and are replacing chemical synthesis [21]. One of these biotechnological processes employs *Bacillus *(*B*.) *subtilis *as the riboflavin cell factory and much work has been carried out in characterising the vitamin's biosynthetic pathway in this organism (For a review see [22]). For *B. subtilis *and *Lactococcus *(*L*.) *lactis *it has been shown that mutants that are isolated on the basis of their resistance to the toxic riboflavin analogue roseoflavin also exhibit a riboflavin-overproduction phenotype [23,24]. Recently, it was demonstrated that fermented dairy products produced either with a roseoflavin-resistant strain of *P. freudenreichii *or *L. lactis*, was able to improve growth and riboflavin status of riboflavin-depleted animals [25,26].

The current study reports on the isolation of roseoflavin-resistant mutants of various strains used in the food industry and analysis of their resulting riboflavin-overproducing phenotype. In the case of *Lb. plantarum *and *Lc. mesenteroides *the mutations responsible for riboflavin overproduction were identified and the possible effects of these mutations on transcription of the *rib *operon are discussed. In the case of *P. freudenreichii *the riboflavin-overproducing strains were examined in comparison to a control strain in a yoghurt production model.

## Results

### Isolation of roseoflavin resistant Leuconostoc mesenteroides

**Figure 1:**
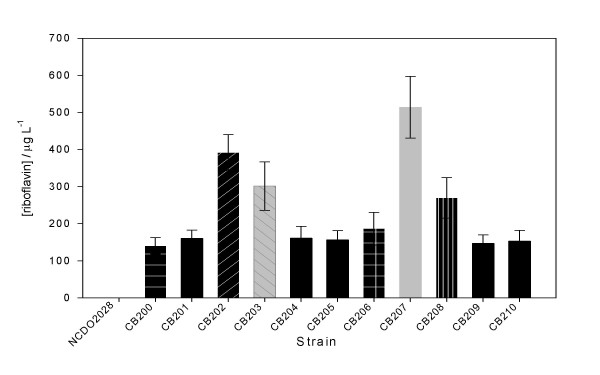
Riboflavin produced by roseoflavin-resistant *Lc. mesenteroides *NCDO2028 mutants. The riboflavin concentration was determined in the cell free supernatant as the cells entered stationary phase. The various patterns indicate different mutations.

It has previously been shown in *B. subtilis *and *L. lactis *that spontaneous resistance to the toxic riboflavin analogue, roseoflavin, frequently coincides with a riboflavin-overproducing phenotype [24,27]. In order to determine whether this method was also applicable to *Lc. mesenteroides*, a wildtype strain of this species was plated at mid exponential growth phase on CDM containing 100 mg/L roseoflavin. In this way a total eleven mutants were isolated. These isolates were grown in CDM and as the cells entered the stationary phase the cell free supernatant was analysed for riboflavin content. All eleven roseoflavin-resistant mutants were found to be riboflavin overproducers (Fig. 1), leading to accumulation of the vitamin into the medium although at clearly different levels ranging from 120 to 500 μg/L.

### Identification of mutations resulting in riboflavin overproduction

**Figure 2:**
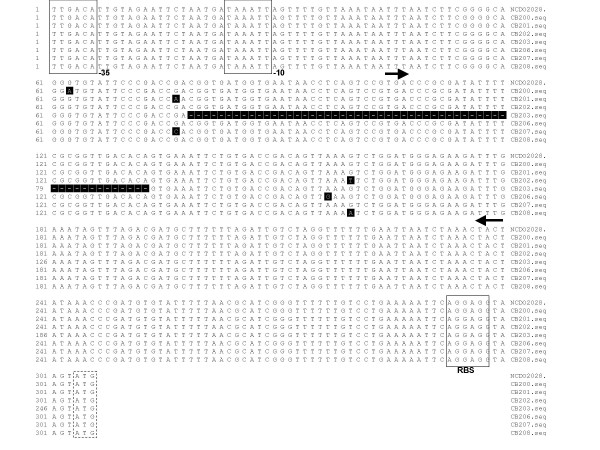
Alignment of the *rib *operon regulatory region of *Lc. mesenteroides *NCDO2028 and seven different mutants isolated. The mutations are shaded. The predicted -10 and -35 recognition sequences and ribosomal binding site are boxed. The *ribG *start codon is boxed with dashed lines. The RFN element predicted by RFAM is indicated by the arrows below the sequence.

*B. subtilis *roseoflavin-resistant mutants have been shown to carry mutations either in the regulatory region of the *rib *operon or in *ribC*, encoding the flavokinase/FAD synthetase responsible for the conversion of riboflavin to its active derivatives FMN and FAD [28-30]. Roseoflavin resistance in *L. lactis *seems to be due to mutations that reside exclusively in the regulatory region upstream of the *rib *operon [24]. It has been found that this region acts as a riboswitch causing premature transcription termination of the *rib *operon in the presence of FMN [31]. The RFN element is a highly conserved domain found within this 5'-untranslated region consisting of 5 stem-loop structures [32]. A predicted RFN element was identified upstream of the *Lc. mesenteroides rib *operon using RFAM (indicated in Fig. 2). To investigate whether mutations were present in the homologous DNA regions in the roseoflavin-resistant *Lc. mesenteroides *mutants chromosomal DNA was isolated from each mutant after which the relevant regions were amplified by PCR and subjected to sequence analysis. No mutations were identified in the *ribC *gene, whereas in each of the isolated mutants the *rib *leader region [Genbank: DQ645591] was shown to contain a mutation (Fig. 2). In only one case (CB203) the mutation was represented by a deletion, while the other ten strains were shown to possess point mutations at one of four different locations within the *rib *leader region. At two of these locations different mutations occurred in different mutants (For example, the *rib *leader region of mutant CB201 contains a G to A substitution at position 77, while strain CB207 contains a G to C substitution at the corresponding position).

### Isolation of riboflavin-overproducing Lb. plantarum and identification of mutations resulting in riboflavin overproduction

**Figure 3:**
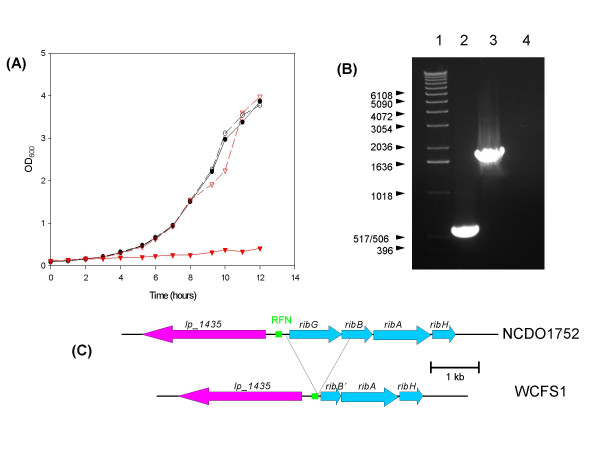
(**A**) Growth of *Lb. plantarum *NCDO 1752 (black lines with circles) and *Lb. plantarum *WCFS1 (red lines with triangles) in CDM (solid lines, closed symbols) or CDM + 5 μM riboflavin (dashed lines, open symbols) (**B**) Amplification of the region from *lp_1345 *to the C terminal of *ribB *from *Lb. plantarum *strains chromosomal DNA. Lane 1: Molecular weight marker; Lane 2: *Lb. plantarum *WCFS1; Lane 3: *Lb. plantarum *NCDO 1752; Lane 4: negative control (H_2_O). The size of the marker bands are indicated to the left of the picture. (**C**) Schematic diagram of the deleted region in *Lb. plantarum *WCFS1 in comparison to *Lb. plantarum *NCDO 1752.

**Figure 4:**
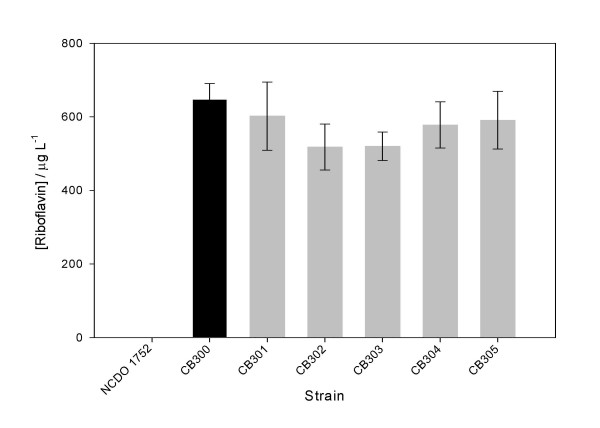
Riboflavin produced by roseoflavin-resistant *Lb. plantarum *NCDO1752 mutants. The riboflavin concentration was determined in the cell free supernatant after the cells entered stationary phase. The two shades represent the two mutants isolated.

**Figure 5:**
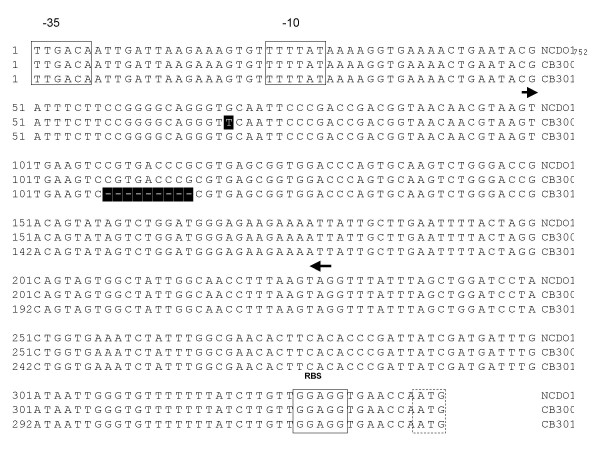
Alignment of the *rib *operon regulatory region of *Lb. plantarum *NCDO1752 and the two mutants isolated. The mutations are shaded. The predicted -10 and -35 recognition sequences and ribosomal binding site are boxed. The *ribG *start codon is boxed with dashed lines. The RFN element predicted by RFAM is indicated by the arrows below the sequence.

Bioinformatic analysis had previously shown that the sequenced strain of *Lb. plantarum *WCFS1 contains an incomplete *rib *operon, in which the entire *ribG *and part of *ribB *are absent from the genome [24,33]. As expected, this strain is unable to grow in the absence of riboflavin (Fig. 3(A)), in contrast to *Lb. plantarum *NCDO1752, which is capable of growth in the absence of the vitamin, thus indicating that this strain encodes a complete and functional riboflavin biosynthetic capability. The latter strain was exposed to 100 mg/L roseoflavin resulting in the isolation of six resistant mutants, which were also shown to be riboflavin overproducers (Fig. 4). In order to analyse the possible mutations in these mutants it was necessary to obtain the sequence of the region upstream of *ribG*, which is not present in the sequenced strain. PCR and sequence analysis of this region from NCDO1752 suggests that the WCFS1 genome harbours a deletion of approximately 1350 base pairs corresponding to the entire *ribG *gene, and part of *ribB *(Figs. 3(B) and 3(C)). The sequence of the regulatory region in NCDO1752 was determined [Genbank: DQ645592], allowing the identification of the putative RFN element using RFAM. This region was then amplified by PCR from the riboflavin-overproducing mutants and their sequence was determined. Only two types of mutations were found (Fig. 5). One strain (CB300) contained a G to T substitution, while the remaining strains (CB301 to 305) contained a 9 base pair deletion within the RFN element.

### Isolation of roseoflavin-resistant Propionibacterium freudenreichii

**Figure 6:**
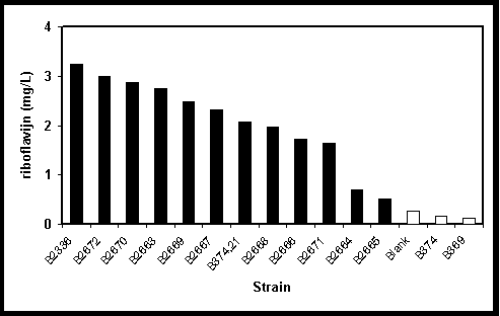
Riboflavin produced by roseoflavin-resistant *P. freudenreichii *B374 mutants. The riboflavin concentration was determined in the cell free supernatant after the cells entered stationary phase. The solid bars represent the levels produced by the roseoflavin-resistant strains, while the open bars represent the wildtype strain concentrations.

Two different strains of *P. freudenreichii *were selected in order to investigate whether the roseoflavin-resistance strategy for the isolation of riboflavin overproducers could also be applied to a representative species of a high-GC gram-positive bacterium. The method was indeed successful as shown by the isolation of twelve riboflavin over-producing mutants of this species, each varying in the amount of riboflavin produced (Fig. 6). Since the genome sequence of *P. freudenreichii *is not publicly available and the amplification of the *rib *leader region of this species using degenerate primers based on the *rib *leader sequence of *P. acnes *was unsuccessful, it was not possible to determine the nature of the mutation(s) resulting in the altered phenotype.

### Stability study of riboflavin overproducing phenotype in *P. freudenreichii*

**Figure 7:**
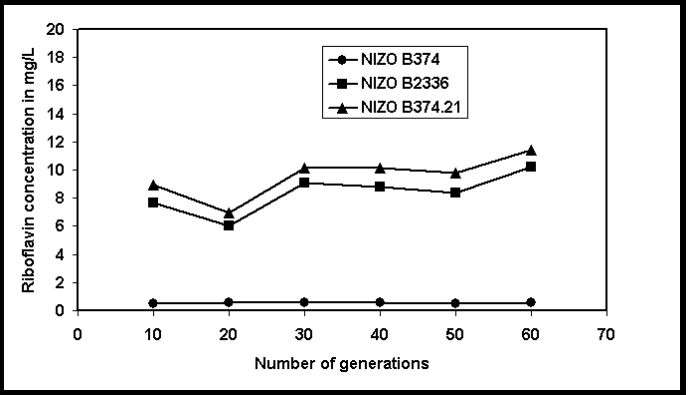
Riboflavin produced by *P. freudenreichii *B374 and its riboflavin overproducing derivatives B2336 and B374.21 after successive generations in roseoflavin free broth.

In order for any of the strains isolated in this study to have potential industrial usefulness it is necessary that the riboflavin-overproducing phenotype is stably maintained. To determine if this is the case, *P. freudenreichii *NIZO B374 and two of its riboflavin-overproducing derivatives were subcultured for sixty generations in the absence of the riboflavin analogue roseoflavin and extracellular riboflavin was determined throughout. It was found that the riboflavin-overproducing phenotype is stably maintained in the absence of the selection pressure for at least 60 generations (Fig. 7).

### Yoghurt study using P. freudenreichii B374 and its riboflavin-overproducing derivative B2336

**Figure 8:**
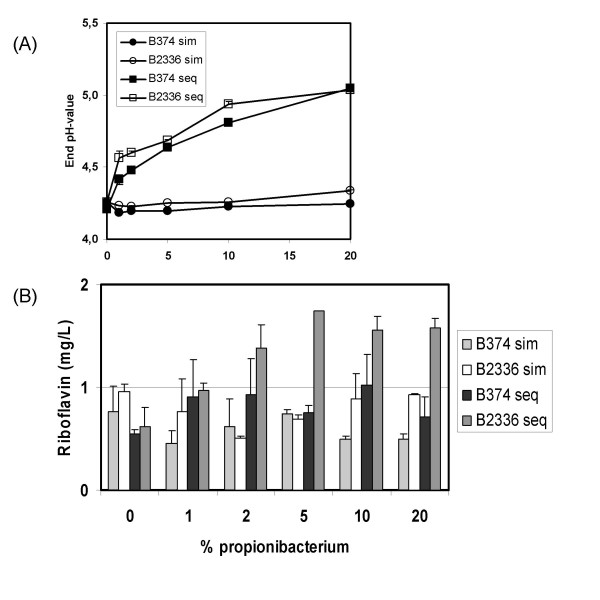
(**A**) Final pH of yoghurt with various inoculum levels of *P. freudenreichii *B374 or B2336 either added 24 hours beforehand or simultaneously with the yoghurt starter culture. (**B**) Final riboflavin levels in yoghurt with various inoculum levels of *P. freudenreichii *B374 or B2336 either added sequentially or simultaneously with the yoghurt starter culture.

In order to determine if the cultures isolated in this study could have industrial potential, a yoghurt production trial was set up, which compared the use of *P. freudenreichii *NIZO B374 and its riboflavin-overproducing derivative *P. freudenreichii *NIZO B2336. To optimize the effect of propionibacteria in a yoghurt fermentation, we compared two different fermentation processes. The effect of co-inoculation of the propionibacteria with the yoghurt starter culture was compared with pre-fermentation by the propionibacteria prior to the addition of the yoghurt starter culture. The viable counts of propionibacteria, streptococci and lactobacilli were unaffected by the presence of the wildtype or riboflavin-overproducing propionibacterial strain. Also sequential inoculation or co-inoculation was did not affect viable counts of the different strains in the starter culture (data not shown). The final pH after fermentation is an important parameter in the yoghurt production process. Figure 8(A) shows the final pH value of the yoghurt at various inoculum levels using both the wildtype and riboflavin-overproducing strains in both sequential and simultaneous inoculation. Both strains have the same effect on acidification of the yoghurt. However, a marked difference is visible between the types of inoculation. Preculturing with propionibacteria counteracts acidification, while no effect was observed when the propionibacteria were added simultaneously with the starter culture. Figure 8(B) shows the final riboflavin concentration of the yoghurt at various inoculum levels of *P. freudenreichii *NIZO B374 and its riboflavin-overproducing derivative *P. freudenreichii *NIZO B2336. Co-inoculation of the propionibacteria with the starter culture has little impact on final riboflavin levels. However, addition of *P. freudenreichii *NIZO B2336 in the sequential fermentation process shows a doubling of the concentration of riboflavin in the fermented end-product.

## Discussion

The work presented in this study demonstrates the broad applicability of the strategy to isolate strains with enhanced vitamin B2 production characteristics using sub-inhibitory levels of roseoflavin. The resultant strains are made through a non-recombinant method and may therefore be readily applicable in an industrial setting. Various biotechnological processes have been developed for industrial scale riboflavin biosynthesis using the ascomycetes *Ashbya gossypii*, different yeasts and the bacterium *B. subtilis *[21]. The latter organism has been successfully employed at a commercial scale to produce riboflavin for feed and food fortification purposes. However, since this production procedure involved a genetically recombinant organism regulatory approval was required where substantial equivalence of the product to non-recombinant riboflavin had to be established, while no DNA from the production strain was allowed to be present in the final product [34]. The purpose of the present study was not to develop strains which would challenge these already available processes, but rather to look at the potential to isolate strains with an improved riboflavin production phenotype that could replace the riboflavin-consuming parent strains in traditional food fermentation processes thus improving the bioavailability of riboflavin.

The *Lc. mesenteroides *strain, NCDO 2028 used in this study was originally isolated from beetroot silage, and was chosen as a representative of a species, which has many varied industrial applications in the dairy industry and various plant and vegetable fermentation processes [35-37]. *Lb. plantarum*, used in a large number of fermentation processes was chosen as another candidate. This organism is found in many diverse environments, owing to its metabolic flexibility [38]. In general, descriptions of the strain's nutritional requirements indicate that riboflavin is not required for growth, indicative of a functional riboflavin biosynthetic pathway [39]. *Lb. plantarum *NCDO 1752, a pickled cabbage isolate, is capable of growth in the absence of the vitamin illustrating its ability to synthesise the vitamin. However, in the course of this work it was revealed that the sequenced strain of *Lb. plantarum*, WCFS1 does not contain a functional *rib *operon and consequently is unable to grow in the absence of exogenous riboflavin [40]. Two strains of *P. freudenreichii *(both isolated from cheese), one subspecies *freudenreichii *(NIZO B374) and one subspecies *shermanii *(NIZO B369) were selected from the NIZO culture collection and the resistance strategy was applied also to this strain. All isolated mutants from these two subspecies were shown to be riboflavin overproducers. The successful application of the roseoflavin resistance strategy in three diverse bacterial species shows that it is a readily employable system in an industrial setting to isolate starter strains that produce and secrete vitamin B2. This is particularly appealing when one considers that some yoghurt cultures have been shown to actually decrease the concentration of riboflavin in some products due to their consumption of the vitamin [41]. In a simultaneously performed collaborative study it was demonstrated that a fermented dairy product produced with *P. freudenreichii *B2336 was able to improve growth and riboflavin status of riboflavin-depleted animals [25]. This proves that the riboflavin produced in yoghurt fermentation is available as a nutrient.

Although not all spontaneous or induced mutations causing riboflavin overproduction in *B. subtilis *have been analysed in detail, the characterised roseoflavin-induced mutations in this organism have been located either in *ribC*, the bifunctional flavokinase/FAD synthetase, which converts riboflavin to FMN and FAD [28,30], or in the regulatory region upstream of the *rib *operon [29]. This knowledge facilitated the identification of the mutations present in the roseoflavin-resistant LAB strains. No mutations were identified in the *ribC *homologue of any of the roseoflavin-resistant strains, but instead all characterised mutants were found to contain mutations in the regulatory leader region upstream of the *rib *operon. In both species different point mutations were identified, which were shown to differentially affect the level of riboflavin overproduction. Additionally, for both LAB species spontaneous roseoflavin-resistant mutants were isolated containing various deletions in the regulatory region of the *rib *operon. It is expected that such mutations are extremely stable. Furthermore, in *P. freudenreichii *it was shown that after sixty generations in non-selective media the riboflavin-overproducing phenotype does not revert to the wildtype phenotype. In analogy to what is known for the regulation of riboflavin biosynthesis in *B. subtilis *and *L. lactis *[24,31,42] it is assumed that also in the species used in this study regulation of the *rib *operon would be mediated by a termination-antitermination mechanism resulting from two different folding options of the RFN element upstream of the operon in response to riboflavin or FMN. RFN elements have been identified in the *rib *operon leader region of *Lc. mesenteroides *(Fig. 2) and *Lb. plantarum *(Fig. 5) using RFAM [43]. It is likely from the position of the mutations that they affect the stability of the terminator structure making it less energetically favourable for it to form, thus allowing continued transcription of the *rib *operon.

In order to study the potential usefulness of such strains in an industrial setting a pilot yoghurt trial was set up to compare addition of *P. freudenreichii *NIZO B374 or its riboflavin-overproducing derivative B2336 either 24 hours prior to the addition of the starter culture or simultaneously with the starter culture. Sequential addition of strain NIZO B2336 to the yoghurt was found to double the concentration of riboflavin in the final product in comparison to yoghurt containing the non-producing wildtype propionibacterium. Furthermore, the mutant showed no differences in comparison to the wildtype strain regarding final cell numbers of the starter culture or final pH of the product. This illustrates that there is a clear benefit of using such riboflavin-overproducing strains in fermentations as it increases the vitamin content of the final product, thus making it more appealing to consumers. Sequential inoculation of the propionibacteria was also found to counteract acidification resulting in a milder product, which could be used as another positive selling point.

The basic concepts of nutrition are changing. The traditional idea of an 'adequate diet', which provides enough nutrients to ensure the individual's survival and meet metabolic needs as well as satisfying hunger is now obsolete. More and more emphasis is being placed on the need for foods to promote health, improve well being and reduce the risk of illness through the adoption of the concept of an "optimum diet" [2]. Selection of strains that have been subjected to uncontrolled genetic alterations has been used in the dairy industry to improve certain intrinsic characteristics of the fermented end product. For example, a spontaneous IS element mediated deletion of the *lacZ *gene altered lactose metabolism resulting in a decreased fermentation of the sugar. Yoghurt made using this strain is not affected by further acidification [44]. Strains modified by induced mutations are considered non-GMO and are acceptable for deliberate release in the European Union [45]. Such strategies could have important implications for food fermentations as industry constantly strives to increase the marketability of their products to more health conscious consumers in an increasingly competitive market.

## Methods

### Bacterial strains, media and culture conditions

The bacterial strains and plasmids used in this study are listed in Table 1. *Lc. mesenteroides *and *Lb. plantarum *were routinely grown in MRS medium [46] or in chemically defined medium (CDM) (adapted by removal of folic acid, riboflavin and nucleotides) [47,48]. *P. freudenreichii *strains were routinely cultured in Lactate Broth (LB) containing tryptone (5 g/L), yeast extract (10 g/L), Na-acetate.3H_2_O (8 g/L) and Na-lactate molasses 60% (25 g/L) at 30°C. The pH of the medium was adjusted to pH 6.5 with 1N KOH before heat treatment for 15 min. at 121°C. The chemically defined medium (CDM) described by Jan *et al*. [49] was modified for the selection of roseoflavin-resistant mutants. The following ingredients were omitted: *p*-aminobenzoic acid, folic acid, riboflavin, L-phenylalanine, L-tryptophane, and L-tyrosine. Enumeration of propionibacteria was done on lactate broth agar plates.

**Table 1:** Strains and plasmids used in this study

**Strains and plasmids**	**Details**	**Reference**
Strains		
*Lc. mesenteroides *NCDO2028	Beetroot silage isolate	UCC culture collection
*Lc. mesenteroides *CB200 to CB210	NCDO2028 derivatives that are resistant to roseoflavin	This study
*Lb. plantarum *WCFS1	Sequenced strain	[33]
*Lb. plantarum *NCDO1752	Pickled cabbage isolate	UCC culture collection
*Lb. plantarum *CB300 to CB305	NCDO1752 derivatives that are resistant to roseoflavin	This study
*P. freudenreichii *NIZO B374	CBS115915, formerly subsp. shermanii, now subsp. freudenreichii; cheese isolate	NIZO culture collection
*P. freudenreichii *NIZO B2336	Roseoflavin resistant derivative of strain NIZO B374	This study
*P. freudenreichii *NIZO B374.21	Roseoflavin resistant derivative of strain NIZO B374	This study
*P. freudenreichii *NIZO B369	CBS115914; From Eidg. Milchw. Versuchsanstalt. Liebefeld	NIZO culture collection
*P. freudenreichii *NIZO B2663 to NIZO B2672	Roseoflavin resistant derivatives of strain NIZO B369	This study

### Isolation of roseoflavin-resistant mutants and sequence analysis of roseoflavin-resistant mutants

Spontaneous roseoflavin-resistant mutants of *Lb. plantarum *and *Lc. mesenteroides *were isolated by plating mid logarithmic phase cells on CDM containing 100 mg l^-1 ^roseoflavin. Isolation of chromosomal DNA from *Lc. mesenteroides *and *Lb. plantarum *was performed as described by Leenhouts *et al*. [50,51]. In order to identify the mutations that were the probable cause of roseoflavin resistance and riboflavin overproduction the regulatory region upstream of *ribG *was amplified by PCR, purified using the JETquick PCR purification kit (Genomed, Löhne, Germany) and subjected to sequence analysis (MWG Biotech AG, Ebersberg, Germany). Spontaneous roseoflavin resistant mutants of *P. freudenreichii *strains were isolated by plating cultures on adapted chemically defined medium (see above) on solid medium made of the same chemically defined medium supplemented with 1% agar and 10 mg/L roseoflavin. After prolonged incubation at 30°C, roseoflavin-resistant colonies were isolated from the plates and further cultured on liquid chemically defined medium supplemented with 10 mg/L roseoflavin. Fully grown cultures were subsequently diluted in fresh medium in which the roseoflavin concentration was stepwise increased from 50 mg/L, then to 100 mg/L and finally to 200 mg/L. From the final culture with 200 mg/L roseoflavin, single colonies were isolated, and analysed for riboflavin production.

### Bioinformatics

In *Lc. mesenteroides *and *Lb. plantarum *potential RFN elements were identified using RFAM, which is a collection of multiple sequence alignments and covariance models representing non-coding RNA families [43,52].

### Quantitative analysis of riboflavin in culture medium

Extracellular riboflavin concentrations were measured by reverse phase HPLC. A Ultrasphere RP 4.6 mm × 25 cm column (Beckman Coulter, Fullerton, CA) was used and riboflavin was eluted with a linear gradient of acetonitrile from 3.6% to 30% at pH 3.2. Fluorescent detection was used and the excitation and emission wavelengths were 440 and 520 nm, respectively. Commercially obtained riboflavin and FMN were used as references and to obtain a standard curve for quantitative purposes (Sigma, Steinheim, Germany).

### Stability study of riboflavin overproducing phenotype in P. freudenreichii

*P. freudenreichii *B374 and its riboflavin-overproducing derivatives were subcultured in lactate broth lacking roseoflavin for 60 generations and extracellular riboflavin levels were measured every 10 generations to determine the stability of the riboflavin-overproducing phenotype.

### Yoghurt study using P. freudenreichii NIZO B374 and its riboflavin-overproducing derivative P. freudenreichii NIZO B2336

Two different methods were applied to produce yoghurt with propionibacteria. First the pasteurized milk was fermented by simultaneous addition of a traditional yoghurt starter culture NIZO S737 (inoculation level of 0.2%) and *Propionibacterium freudenreichii *NIZO B374 (wildtype) or *P. freudenreichii *NIZO B2336 (riboflavin-overproducing derivative of strain NIZO B374). The inoculated yoghurt milk was subsequently incubated for 16 hours at 30°C. *P. freudenreichii *NIZO B374 or NIZO B2336 were added at various inoculum levels (0, 1, 2, 5, 10 or 20%). Secondly, the pasteurized milk was pre-fermented for 24 hours at 30°C with the propionibacteria (inoculum levels of 0, 1, 2, 5, 10 or 20%), before addition of the yoghurt starter culture. The second phase of fermentation was again 16 hours at 30°C. At the end of fermentation the pH and viable counts were determined and the riboflavin content of the yoghurt was measured.

### Nucleotide sequence accession numbers

The nucleotide sequence data of the 5' leader regions upstream of the riboflavin biosynthesis operons in *Lc. mesenteroides *NCDO 2028 and *Lb. plantarum *NCDO 1752 reported in this paper have been submitted to the Genbank database under accession numbers DQ645591 and DQ645592 respectively.

## Competing interests

The author(s) declare that they have no competing interests.

## Authors' contributions

CB performed all work and analyses on *Lc. mesenteroides *and *Lb. plantarum *including the isolation of the mutants, bioinformatic analysis and identification of mutations. CB also drafted the manuscript. GR performed all the work with *P. freudenreichii *including isolation of the mutants, the stability assay and the yoghurt trial. DvS and ES participated in the design of the study and the coordination and supervision of the work. DvS and ES also helped to draft the manuscript. All authors read and approved the final manuscript.

## References

[B1] Kalra EK (2003). Nutraceutical-Definition and Introduction. AAPS Pharm Sci.

[B2] Palou A (2004). Food Safety and Functional Foods in the European Union: Obesity as a Paradigmatic Example for Novel Food Development. Nutr Rev.

[B3] Sanders ME (1998). Overview on functional foods: emphasis on probiotic bacteria. Int Dairy J.

[B4] Stanton C, Ross RP, Fitzgerald GF, Sinderen DV (2005). Fermented functional foods based on probiotics and their biogenic metabolites. Current Opinion in Biotechnology.

[B5] Stanton C, Gardiner G, Meehan H, Collins K, Fitzgerald GF, Lynch PB, Ross RP (2001). Market potential for probiotics. Am J Clin Nutr.

[B6] Metchnikoff E (1908). The prolongation of life.

[B7] Kleerebezem M, Hugenholtz J (2003). Metabolic pathway engineering in lactic acid bacteria. Curr Opin Biotech.

[B8] Hugenholtz J, Smid EJ (2002). Nutraceutical production with food-grade organisms. Curr Opin Biotech.

[B9] Thierry A, Maillard MB, Herve C, Richoux R, Lortal S (2004). Varied volatile compounds are produced by Propionibacterium freudenreichii in Emmental cheese.. Food Chem.

[B10] Gardner N, Champagne CP (2005). Production of Propionibacterium shermanii biomass and vitamin B12 on spent media. J Appl Microbiol.

[B11] Huang Y, Adams MC (2004). In vitro assessment of the gastrointestinal tolerance of potential probiotic dairy propionibacteria.. Int J Food Microbiol.

[B12] Blanck HM, Bowman BA, Serdula MK, Khan LK, Kohn W, Woodruff BA (2002). Angular stomatitis and riboflavin status among adolescent Bhutanese refugees living in southeastern Nepal. Am J Clin Nutr.

[B13] McKinley MC, McNulty H, McPartlin J, Strain JJ, Scott JM (2002). Effect of riboflavin supplementation on plasma homocysteine in elderly people with low riboflavin status. Eur J Clin Nutr.

[B14] Langohr HD, Petruch F, Schroth G (1981). Vitamin B 1, B 2 and B 6 deficiency in neurological disorders. J Neurol.

[B15] O'Brien MM, Kiely M, Harrington KE, Robson PJ, Strain JJ, Flynn A (2001). The North/South Ireland Food Consumption Survey: vitamin intakes in 18-64-year-old adults. Public Health Nutr.

[B16] Baku TK, Dickerson JWT (1996). Vitamins in Human Health and Disease.

[B17] Boehnke C, Reuter U, Flach U, Schuh-Hofer S, Einhaupl KM, Arnold G (2004). High-dose riboflavin treatment is efficacious in migraine prophylaxis: an open study in a tertiary care centre. Eur J Neurol.

[B18] Akompong T, Ghori N, Haldar K (2000). In vitro activity of riboflavin against the human malaria parasite Plasmodium falciparum. Antimicrob Agents Chemother.

[B19] Coimbra CG, Junqueira VB (2003). High doses of riboflavin and the elimination of dietary red meat promote the recovery of some motor functions in Parkinson's disease patients. Braz J Med Biol Res.

[B20] Cooperman JM, Lopez R, Machlin LJ (1991). Riboflavin. Handbook of Vitamins.

[B21] Stahmann KP, Revuelta JL, Seulberger H (2000). Three biotechnical processes using Ashbya gossypii, Candida famata, or Bacillus subtilis compete with chemical riboflavin production. Appl Microbiol Biotechnol.

[B22] Perkins JB, Pero J, Sonenshein A, Hoch J and Losick R (2002). Vitamin Biosynthesis. Bacillus subtilis and Its Closest Relatives: from Genes to Cells.

[B23] Kukanova AI, Zhdanov VG, Stepanov AI (1982). Bacillus subtilis mutants resistant to roseoflavin. Genetika.

[B24] Burgess C, O' Connell-Motherway M, Sybesma W, Hugenholtz J, van Sinderen D (2004). Riboflavin production in Lactococcus lactis: potential for in situ production of vitamin-enriched foods?. Appl Environ Microbiol.

[B25] LeBlanc JG, Rutten G, Bruinenberg P, Sesma F, Savoy de Giori G, Smid EJ (2006). A novel dairy product fermented with Propionibacterium freudenreichii improves the riboflavin status of deficient rats. Nutrition.

[B26] LeBlanc JG, Burgess C, Sesma F, Savoy de Giori G, van Sinderen D (2005). Ingestion of milk fermented by genetically modified Lactococcus lactis improves the riboflavin status of deficient rats.. J Dairy Sci.

[B27] Perkins JB, Pero J, Sloma A (1991). Riboflavin overproducing strains of bacteria.

[B28] Kreneva RA, Perumov DA (1990). Genetic mapping of regulatory mutations of Bacillus subtilis riboflavin operon. Mol Gen Genet.

[B29] Kil YV, Mironov VN, Gorishin IY, Kreneva RA, Perumov DA (1992). Riboflavin operon of Bacillus subtilis: unusual symmetric arrangement of the regulatory region. Mol Gen Genet.

[B30] Coquard D, Huecas M, Ott M, van Dijl JM, van Loon AP, Hohmann HP (1997). Molecular cloning and characterisation of the ribC gene from Bacillus subtilis: a point mutation in ribC results in riboflavin overproduction. Mol Gen Genet.

[B31] Winkler WC, Cohen-Chalamish S, Breaker RR (2002). An mRNA structure that controls gene expression by binding FMN. Proc Natl Acad Sci U S A.

[B32] Gelfand MS, Mironov AA, Jomantas J, Kozlov YI, Perumov DA (1999). A conserved RNA structure element involved in the regulation of bacterial riboflavin synthesis genes. Trends Genet.

[B33] Kleerebezem M, Boekhorst J, van Kranenburg R, Molenaar D, Kuipers OP, Leer R, Tarchini R, Peters SA, Sandbrink HM, Fiers MW, Stiekema W, Lankhorst RM, Bron PA, Hoffer SM, Groot MN, Kerkhoven R, de Vries M, Ursing B, de Vos WM, Siezen RJ (2003). Complete genome sequence of Lactobacillus plantarum WCFS1. Proc Natl Acad Sci U S A.

[B34] Bretzel W, Schurter W, Ludwig B, Kupfer E, Doswald S, Pfister M, van Loon AP (1999). Commercial riboflavin production by recombinant Bacillus subtilis: down-stream processing and comparison of the composition of riboflavin produced by fermentation or chemical synthesis. J Ind Microbiol Biotech.

[B35] Coulin P, Farah Z, Assanvo J, Spillmann H, Puhan Z (2006). Characterisation of the microflora of attieke, a fermented cassava product, during traditional small-scale preparation.. Int J Food Microbiol.

[B36] Plengvidhya V, Breidt F, Fleming HP (2004). Use of RAPD-PCR as a method to follow the progress of starter cultures in sauerkraut fermentation.. Int J Food Microbiol.

[B37] Stiles ME, Holzapfel WH (1997). Lactic acid bacteria of foods and their current taxonomy.. Int J Food Microbiol.

[B38] Nicoloff H, Arsene-Ploetze F, Malandain C, Kleerebezem M, Bringel F (2004). Two arginine repressors regulate arginine biosynthesis in Lactobacillus plantarum.. J Bacteriol.

[B39] Hammes WP, Wood BJB and Holzapfel WH (1995). The genus Lactobacillus. The Genera of Lactic Acid Bacteria.

[B40] Teusink B, van Enckevort FHJ, Francke C, Wiersma A, Wegkamp A, Smid EJ, Siezen R (2005). In silico reconstruction of the metabolic pathways of Lactobacillus plantarum: Comparing predictions of nutrient requirements with those from growth experiments.. Appl Environ Microbiol.

[B41] Elmadfa I, Heinzle C, Majchrzak D, Foissy H (2001). Influence of a probiotic yoghurt on the status of vitamins B(1), B(2) and B(6) in the healthy adult human. Ann Nutr Metab.

[B42] Mironov AS, Gusarov I, Rafikov R, Lopez LE, Shatalin K, Kreneva RA, Perumov DA, Nudler E (2002). Sensing small molecules by nascent RNA: a mechanism to control transcription in bacteria. Cell.

[B43] Griffiths-Jones S, Bateman A, Marshall M, Khanna A, Eddy SR (2003). Rfam: an RNA family database. Nucleic Acids Res.

[B44] Mollet B, Delley M (1990). Spontaneous deletion formation within the beta-galactosidase gene of Lactobacillus bulgaricus. J Bacteriol.

[B45] European Union Council (2001). Directive on the deliberate release into the environment of genetically modified organisms. 2001/18/EC.

[B46] de Man JC, Rogosa M, Sharp ME (1960). A medium for the cultivation of Lactobacilli. J Appl Bacteriol.

[B47] Otto R, ten Brink B, Veldkamp H, Konings WN (1983). The relation between growth rate and electrochemical gradient in Streptococcus cremoris.. FEMS Microbiol Lett.

[B48] Poolman B, Konings WN (1988). Relation of growth of Streptococcus lactis and Streptococcus cremoris to amino acid transport. J Bacteriol.

[B49] Jan G, Leverrier P, Pichereau V, Boyavall P (2001). Changes in Protein Synthesis and Morphology during Acid Adaptation of Propionibacterium freudenreichii. Appl Environ Microbiol.

[B50] Leenhouts KJ, Kok J, Venema G (1989). Campbell-like integration of heterologous plasmid DNA into the chromosome of Lactococcus lactis subsp. lactis. Appl Environ Microbiol.

[B51] Leenhouts KJ, Kok J, Venema G (1991). Replacement recombination in Lactococcus lactis. J Bacteriol.

[B52] RFAM (RNA family database). http://www.sanger.ac.uk/Software/Rfam/.

